# Tamsulosin Associated with Interstitial Lung Damage in CYP2D6 Variant Alleles Carriers

**DOI:** 10.3390/ijms21082770

**Published:** 2020-04-16

**Authors:** Naomi T. Jessurun, Petal A. Wijnen, Aalt Bast, Eugène P. van Puijenbroek, Otto Bekers, Marjolein Drent

**Affiliations:** 1 Scientific Department, Netherlands Pharmacovigilance Centre Lareb, 5237 MH’s-Hertogenbosch, The Netherlands; 2 ild Care Foundation Research Team, 6711 NR Ede, The Netherlands; 3 Department of Clinical Chemistry, Central Diagnostic Laboratory, Maastricht University Medical Centre, 6202 AZ Maastricht, The Netherlands; 4 Department of Pharmacology and Toxicology, Faculty of Health, Medicine and Life Sciences, Maastricht University, 6229 ER Maastricht, The Netherlands; 5 Venlo Campus, Maastricht University, 5911 BV Venlo, The Netherlands; 6 Faculty of Science and Engineering, Groningen Research Institute of Pharmacy, University of Groningen, 9747 AG Groningen, The Netherlands; 7 ILD Center of Excellence, St. Antonius Hospital, 3435 CM Nieuwegein, The Netherlands

**Keywords:** tamsulosin, drug-induced interstitial lung disease, pharmacogenetics, CYP2D6, CYP3A4/5, drug metabolizing enzymes, drug metabolites, cytochrome P450

## Abstract

Drugs are serious but underestimated causative agents of interstitial lung disease (ILD). Both cytotoxic and immune mechanisms may be involved in drug-induced ILD (DI-ILD). We aimed to investigate whether polymorphisms of relevant CYP enzymes involved in the metabolization of tamsulosin might explain the pathologic mechanism of the DI-ILD in the cases with suspected tamsulosin DI-ILD. We collected 22 tamsulosin-associated DI-ILD cases at two ILD Expertise Centers in the Netherlands between 2009 and 2020. CYP2D6, CYP2C9, CYP2C19, CYP3A4, and CYP3A5 single nucleotide polymorphisms were genotyped and compared with a control group of 78 healthy Caucasian male volunteers. Nine cases were phenotyped as CYP2D6 poor metabolizers and 13 as CYP2D6 intermediate metabolizers. The phenotypes of the cases differed significantly from those of the healthy controls, with more poor metabolizers. After withdrawal of tamsulosin, the pulmonary condition of three cases had improved, six patients had stabilized, and one patient stabilized after reducing the tamsulosin dose. The described 22 cases suggest that an association between the presence of CYP2D6 allelic variants and tamsulosin-associated ILD is highly likely. These cases highlight the importance of both clinical and genetic risk stratification aimed to achieve a more accurate prevention of DI-ILD in the future and enhance the quality of life of patients.

## 1. Introduction

Nowadays, it is well recognized that genetic polymorphisms in genes coding for enzymes responsible for drug metabolism and drug disposition are of great importance for the efficacy and toxicity of medicines [1]. It is generally agreed that the cytochrome P450 (CYP) superfamily of enzymes, with more than 1000 isoenzymes, five of which (CYP3A4, CYP2D6, CYP2C9, CYP2C19, and CYP1A2) metabolize 90% of all drugs, contributes greatly to the metabolization of drugs in the human body. Identifying polymorphisms of these *CYP*s is mostly done to predict or explain drug target serum levels, e.g., a reduced CYP metabolism leads to increased serum drug levels and to increased toxicity. The drug metabolites formed are sometimes assessed for their pharmacological activity, but the toxic characteristics are rarely acknowledged or recognized.

Drug metabolite toxicity is best illustrated by the example of acetaminophen. It is metabolized by several CYP enzymes to its reactive metabolite, N-acetylparabenzoquinone-imine (NAPQI) which depletes the scavenger glutathione and binds to liver proteins, leading to liver injury. Although the phrase acetaminophen-induced hepatotoxicity is used in the case of intentional auto-intoxications, it is NAPQI that determines the final hepatotoxicity [2].

**Figure 1:**
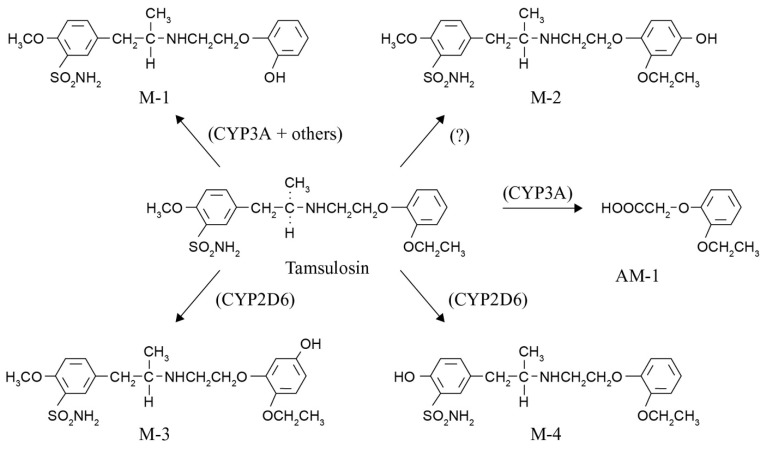
Tamsulosin is metabolized to five known metabolites by cytochrome P450 enzymes, mainly CYP3A4/5 and CYP2D6. After oral administration, M-1 is the major product (15.7% of the dose), followed by AM-1 (7.5% of the dose) and M-3 (6.4% of the dose). The formation of tamsulosin metabolites M-1 and AM-1 is mainly catalyzed by CYP3A isoforms (CYP3A4/5). The formation of M-3 is mainly catalyzed by CYP2D6 [8,9].

Considering *CYP* polymorphisms and adverse drug reactions (ADRs) of expected drug metabolites is not current clinical practice. Even less common is the use of pharmacogenetic knowledge to assess the impact on shifts in drug metabolization pathways and the formation of unexpected toxic metabolites [3]. The potential importance of this assessment is illustrated in this paper by 22 cases of tamsulosin-associated interstitial lung disease (ILD). ILD is a group of heterogeneous disorders that diffusely involve the lung parenchyma. There is an ever-increasing number of drugs that can produce variegated patterns of drug-induced ILD (DI-ILD), and virtually all are histopathologic patterns of interstitial pneumonia. However, drugs are underestimated as serious causative agents of ILD, and elucidating the causative drug is challenging [4,5]. At present, more than 350 drugs are known to cause injury to the lung, and new causative drugs are regularly being identified [6]. Furthermore, previous studies showed that DI-ILD is associated with reduced metabolic capacity [7]. The association between tamsulosin and DI-ILD has not been described before. We aimed to investigate whether polymorphisms of relevant CYP enzymes involved in the metabolization of tamsulosin and the subsequent formation of drug metabolites (Figure 1) might explain the pathologic mechanism of the DI-ILD thus incurred.

## 2. Results

### 2.1. Description of the Cases

Data of 22 male patients who were taking tamsulosin (0.4 mg daily, orally) and developed side-effects were collected during a 12-year period. The patients experienced various manifestations of hypersensitivity while taking the medication. They recalled a history of progressive dyspnoea and exercise limitation for six months to four years prior to the referral, depending on when tamsulosin was started. High resolution computed tomography (HRCT) showed features of either non-specific interstitial pneumonia (NSIP) or idiopathic pulmonary fibrosis (IPF; end-stage pulmonary fibrosis). The diagnosis was confirmed by an experienced radiologist. Other causes of these ILDs were excluded, such as connective tissue diseases related interstitial lung disease (CTD-ILD) and familial IPF (FIPF). In 11 cases, the diagnosis was NSIP, and the other 11 patients were eventually diagnosed with IPF. All patients were male, with a mean age (SD) of 78.5 (6.3) years; range 68–93 (Table 1). The median latency period till diagnosis was six months (range three months to seven years). Eight patients used metoprolol concomitantly, which is also a substrate for CYP2D6. Tamsulosin had been withdrawn in almost all patients. After withdrawal of tamsulosin, the pulmonary condition of three cases (AS 1.0) had improved, six patients had stabilized, and one patient with an AS of 1.0 stabilized after reducing the tamsulosin dose (half of the dosage every other day, see also Table 1). Of the remaining 12 cases, six had died of comorbidities and three (all suffering from IPF) had died of respiratory failure, and of three cases follow-up data were lacking. The outcome for the causality score of the individual cases using the Naranjo probability scale was ‘probable’ in all 22 cases.

### 2.2. Genotyping

Nine patients (41%) were phenotyped as CYP2D6 poor metabolizers (PMs) and 13 patients (59%) as IMs (Table 1). The phenotypes of the cases differed significantly from those of the healthy controls (*p* < 0.001), with particularly more PMs and fewer extensive metabolizers (EMs) than in the controls (Table 2). Seventeen patients were genotyped as CYP3A5 non-expressors (the most common genotype [wild type] in a Caucasian population) and three appeared to be heterozygote expressors, producing functional CYP3A5 enzyme.

## 3. Discussion

This paper is the first to describe a case series of DI-ILD associated with tamsulosin use. The association is supported by a mechanistic approach based on CYP2D6 and CYP3A enzyme metabolism and the formation of metabolites. The possible role of pharmacogenetics is also illustrated by the substantial differences in the *CYP2D6* phenotypes frequencies between the 22 tamsulosin-associated ILD cases and the healthy volunteers.

The CYP2D6 and CYP3A enzymes involved are abundantly expressed in the human liver and lung. Additionally, just as in the liver, the pathogenesis of drug-associated cell injury in the lung may involve immune and cytotoxic mechanisms of action in which pharmacogenetics, reactive oxygen species, and reactive drug metabolites may play a role [10,11].

Considering all the available knowledge on characteristics and polymorphisms of *CYP2D6* and *CYP3A* and the drug metabolization pathway of tamsulosin, two important questions remain to be answered. The first one is whether we could have predicted these tamsulosin-associated ILD cases by applying pharmacogenetics, and the second one is what lesson we can learn from these cases. Knowledge of the *CYP2D6* phenotype or activity score for these patients could have predicted a possible shift in metabolization to the CYP3A pathway, resulting in more of the M-1, M-2, and AM-1 metabolites (see Figure 1). The most toxic metabolite formed is AM-1 (o-ethoxyphenoxy acetic acid), containing a carboxylic acid moiety. Several non-steroidal anti-inflammatory drugs (NSAIDs) such as ibufenac, bromfenac, zomepirac, benoxaprofen, and pirprofen with this carboxylic acid moiety have been withdrawn from the market due to rare, mostly hepatic, ADRs [12,13,14]. A search in the World Health Organization’s Global Individual Case Safety Report database (VigiBase^®^), maintained by the Uppsala Monitoring Centre in Sweden, showed that several reports of ILD have been received for these NSAIDs, indicating that drugs and metabolites with this moiety have previously been associated with the occurrence of ILD. Bioactivation of this carboxyl moiety forms reactive metabolites, namely coenzyme A thioesters and acyl glucuronides, representing an early step in the pathogenesis of ensuing adverse effects. Acyl glucuronides can covalently modify proteins via a simple transacylation reaction, or through an acyl migration within the β-O-glucuronide unit to a reactive R-hydroxy-aldehyde intermediate, which can react with proteins [15].

Acyl-Coenzyme A (acyl-CoAs) thioesthers of the carboxylic acid moieties in drugs possess sufficient electrophilicity for nucleophilic reactions with amino acids and are able to form covalent adducts with proteins. Just like cytochrome P450 enzymes, the cofactor for the acyl glucuronidation, uridinediphospho-glucuronic acid (UDPGA), is mostly expressed in the liver, but also in extra-hepatic tissues such as the skin and the lungs. Although liver and skin reactions are well-known and have previously been related to the bioactivation of the carboxylic moiety in drugs, this is the first publication in which lung reactions have been associated with this moiety. Furthermore, despite their reactivity, these reactive metabolites are sufficiently stable to be transported out of the cell into the circulation. Although the evidence for the formation of these acyl glucuronides, their reaction with proteins and the potential clinically relevant ADRs in vivo is limited, there is a large body of in vitro data. Additionally, it is widely acknowledged that carboxylic drugs and carboxylic drug metabolites are prone to forming reactive metabolites that have the potential to play a mechanistic role in ADRs associated with the therapy concerned [15].

Three patients appeared to be heterozygote expressors of CYP3A5, producing functional CYP3A5, an enzyme abundantly expressed in the lung [10]. This induces a faster metabolization of tamsulosin, but there are no indications that it affects the outcome of the ILD.

Only after all other possible causes have been excluded can the diagnosis of DI-ILD be made [16,17]. However, the differences in CYP2D6 phenotypes between the 22 cases and the controls suggest a role for genetics in the development of tamsulosin-associated ILD. Increased understanding of genetic variants in drug-metabolizing enzymes, followed by stratification based on these genetic variants and their possible relation with the proposed cytotoxic drug metabolites, may offer an opportunity to prevent the often serious DI-ILDs [7]. A lesson to be learnt from this might be that taking full advantage of pharmacogenetics in clinical practice requires more effort and more expertise than is currently being applied.

This deduction from the available knowledge, suggesting a positive association between tamsulosin-associated DI-ILD and decreased CYP2D6 activity, has many limitations. Although tamsulosin was the most suspected drug for the DI-ILD—in that after withdrawal the condition stabilized in most cases and improved in a few—no hard causal relationship can be established between the polymorphisms, the drug, and the DI-ILD. In addition, evidence regarding tamsulosin drug levels is lacking because in our cases of DI-ILD, the suspected drugs were withdrawn before serum levels were determined or conclusions were drawn. According to the Naranjo algorithm, which is still the most widely used causality method, all individually assessed cases were ‘probable’. Unfortunately, no tamsulosin and/or metabolite serum levels were available of the presented cases and rechallenge was not considered, though this would have strengthened our observation. Moreover, we realize that the Naranjo algorithm probably does not cover all types of ADRs and might need adjustments [18,19]. A search for more cases yielded multiple reports of tamsulosin-associated DI-ILD in EudraVigilance (the system for suspected ADRs in the European Economic Area) and in Vigibase^®^, but unfortunately, information on genotyping was lacking for these cases. However, a few patients in the EudraVigilance database concomitantly used CYP2D6 inhibitors such as paroxetine, which reduces the metabolic activity of CYP2D6 even in low doses, and this probably turned these patients into poor or intermediate CYP2D6 metabolizers [20]. So far, no studies have measured the impact of *CYP2D6* and *CYP3A* polymorphisms on the formation of tamsulosin metabolites and cytotoxic reactions. Although the present case series shows a possible important role for pharmacogenetics, drug metabolization pathways and drug metabolites in the development of DI-ILD, it is observational and descriptive. Further research should confirm the suggested relationships.

A ‘one-size-fits all’ approach to drug prescription is based on broad population averages, whereas personalized medicine offers more effective and safer drug therapy that is tailor-made for individual patients. The growing understanding of pharmacogenetics and pharmacogenomics offers many advantages in terms of customizing drug use, which may result in better disease outcomes, less drug wastage, lower drug costs, safer drug prescriptions, and more effective treatments. The case series we present shows that genetic variations in metabolizing enzymes should be considered in the development of DI-ILD. NSIP and IPF are both regarded as chronic interstitial fibrosis or idiopathic interstitial pneumonia (IIP) of unknown cause. Compared to other IIPs, IPF has a significantly worse prognosis. The prognosis of NSIP is variable. Some patients improve, others remain stable or improve on treatment, but some evolve to end stage fibrosis IPF and finally die of the disease [16]. Therefore, it is of great clinical relevance to identify agents likely to be involved in the initiation and/or progression of the fibrotic process. One of these agents/triggers might be drugs, as was the case in our 22 presented cases. In earlier studies we found an association of certain gene variants with the appearance of DI-ILD [7,21,22,23,24,25,26]. This paves the way for a potential use of personalized medicine by genotyping, aiming to improve efficacy, tolerability, and drug safety.

## 4. Methods

### 4.1. Patients and Methods

Patients presented with suspected tamsulosin-associated ILD (either NSIP or IPF (end-stage pulmonary fibrosis)) at the ILD Center of Excellence at St. Antonius Hospital, Nieuwegein, and at the Maastricht University Medical Centre (MUMC), both in the Netherlands (2009–2020), were selected. A multidisciplinary team confirmed the diagnosis based on clinical presentation, including dyspnea and hypoxia, pulmonary function impairment, exercise intolerance, and high-resolution CT-scan abnormalities, including multifocal areas of ground-glass opacity with intralobular interstitial thickening. Other possible causes, such as infections and other drug use, were meticulously excluded. The control group regarding the distribution of allele variants in the general population consisted of 78 healthy Caucasian male volunteers (average age 38 years) who did not use any medication nor had any relevant medical history. All healthy volunteers were MUMC hospital employees [7]. The study was performed in accordance with the Declaration of Helsinki and its amendments. The protocol was approved by the local Medical Ethics Board of the MUMC. The medical ethics review committee of the MUMC approved the study (MAC# - METC 11-4-116, 9 November 2011). Written informed consent for participation in this study was obtained from all subjects. Demographic information of the cases (gender, age), tamsulosin dosage and all available concomitant medication data were gathered from (electronic) patient records. Since the patients had been referred to the two above centers, the tamsulosin treatment had already been stopped or reduced. Hence, as the determination of tamsulosin and/or its metabolites is not standard practice in the Netherlands, no serum drug levels were available or could be obtained. The causality score of the individual cases was assessed using the Naranjo Probability Scale [27].

### 4.2. Genotyping

DNA was obtained from all subjects from venous EDTA-anticoagulated blood. Genotyping of *CYP2D6*, *CYP3A4*, and *CYP3A5* single nucleotide polymorphisms (SNPs) was done by real-time PCR Fluorescence Resonance Energy Transfer (FRET) assays on the LightCycler (Roche Diagnostics, Mannheim, Germany). We used the CYP2C9 and CYP2C19 Mutation Detection Kits (Roche Diagnostics, Mannheim, Germany), and CYP3A4 and CYP3A5 FRET primer-probe mixes (TIB MOLBIOL, Berlin, Germany), according to the manufacturer’s instructions. The *CYP2D6* SNPs were genotyped using the Luminex xTAG CYP2D6 Kit v3 and the LX200 (Luminex, Austin, TX, USA), according to the manufacturer’s instructions.

According to conventional classification systems, individuals were phenotyped as poor metabolizer (PM) if they carried two non-functional alleles; as intermediate metabolizer (IM) if they carried one non-functional allele or two reduced activity alleles; as extensive metabolizer (EM) if they carried one allele associated with reduced activity and one functional allele or two functional alleles, and as ultra-rapid metabolizer (UM) if they carried at least two copies of a functional allele plus a reduced activity allele or three copies of a functional allele.

### 4.3. Statistical Analysis

Statistically significant differences between CYP2D6 phenotype frequencies in cases and controls were assessed using a Fisher exact test in R (version 3.5.1, Vienna, Austria) [28]. A *p*-value less than 0.05 was considered statistically significant.

## 5. Conclusions

Tamsulosin has so far not been recognized in general clinical practice as an agent that might be associated with the development and/or progression of lung damage. Although the sample size is rather low, the described 22 cases suggest that an association between the presence of CYP2D6 allelic variants and tamsulosin-associated ILD is highly likely. We acknowledge that the sample size is rather small as to allow us to calculate an effect size for the CYP2D6 phenotype on the occurrence of tamsulosin-associated ILD. However, the CYP2D6 phenotype of the 22 presented cases differed significantly from that of a control population of healthy male volunteers, in that there were more poor and intermediate metabolizers than among the controls. This may support the idea of an alternative metabolization route via CYP3A and the formation of the reactive metabolite (AM-1), which we associated with lung toxicity. These cases show the importance of including genetic risk stratification (pharmacogenomics) in the work-up of patients with suspected drug-induced (lung) toxicity, and the advantages of genotyping prior to drug prescription. This may be clinically useful for the prediction and prevention of ADRs in general and in our cases for drug-induced pulmonary toxicity, in particular by reducing the risk of development or progression of end-stage pulmonary fibrosis. Furthermore, genotyping and phenotyping drug-metabolizing enzymes prior to prescription has the potential to contribute to safe drug use in patients using multiple drugs. Lack of familiarity with this approach may lead to causative factors being ignored and to unnecessary delays in their recognition. Both clinical and genetic risk stratification may lead to a more accurate prevention of drug-induced lung damage in the future and enhance the quality of life of the patients.

## Figures and Tables

**Table 1:** Characteristics of the 22 patients with suspected tamsulosin-induced ILD. Abbreviations: NSIP = non-specific interstitial pneumonia; IPF = idiopathic pulmonary fibrosis; AS = activity score (range between AS = 0 (poor metabolizer) and AS > 2 (ultra-rapid metabolizer)); n/a = not available; PM = poor metabolizer; IM = intermediate metabolizer.

Patient	Age (Years)	Diagnosis, Condition after Withdrawal of Tamsulosin	Genotype							Concomitant Drugs and the Most Important Metabolizing Cytochrome P450 Isoenzyme for Them		
			*CYP2D6* (including Phenotype)			*CYP2C9*	*CYP3A4*	*CYP3A5*	*CYP2C19*	CYP2D6	CYP2C9	CYP2C19
1	70	NSIP, stabilized	*4/*41	AS: 0.5	(IM)	*1/*2	*1A/*1A	*3/*3	*1/*1			
2	84	NSIP, stabilized	*4/*4	AS: 0.0	(PM)	*1/*2	*1A/*1A	*3/*3	*1/*1	Metoprolol	Valsartan	
3	79	NSIP, stabilized	*4/*6	AS: 0.0	(PM)	*2/*3	*1A/*1A	*3/*3	*1/*1			
4	93	IPF, stabilized	*4/*4	AS: 0.0	(PM)	*1/*1	*1A/*1A	*3/*3	*1/*2	Metoprolol		
5	87	IPF, stabilized	*3/*4	AS: 0.0	(PM)	*1/*1	*1A/*1B	*1/*3	*1/*1			
6	79	IPF, stabilized	*4/*4	AS: 0.0	(PM)	*1/*2	n/a	n/a	*1/*1	Metoprolol		
7	76	IPF, stabilized ^#^	*1/*4	AS: 1.0	(IM)	*1/*2	*1A/*1A	*3/*3	*1/*2			
8	83	NSIP, improved	*1/*4	AS: 1.0	(IM)	*1/*1	n/a	n/a	*1/*1			
9	72	NSIP, improved	*1/*4	AS: 1.0	(IM)	*1/*2	*1A/*1A	*3/*3	*1/*1			
10	77	NSIP, improved	*1/*4	AS: 1.0	(IM)	*1/*1	n/a	*1/*3	*1/*1	Metoprolol		
11	71	IPF, died from pneumonia	*4/*4	AS: 0.0	(PM)	*1/*1	*1A/*1A	*3/*3	*1/*2			
12	79	IPF, died from cardiac failure	*5/*5	AS: 0.0	(PM)	*1/*2	*1A/*1A	*3/*3	*1/*1		Losartan	Clopidogrel
13	79	IPF, died from respiratory failure	*1/*4	AS: 1.0	(IM)	*1/*1	*1A/*1A	*3/*3	*2/*2			
14	68	IPF, died from respiratory failure	*1/*4	AS: 1.0	(IM)	*1/*1	*1A/*1A	*3/*3	*2/*2			
15	74	IPF, died from respiratory failure	*4/*6	AS: 0.0	(PM)	*1/*2	*1A/*1A	*3/*3	*1/*1	Metoprolol	Rosuvastatin	
16	80	NSIP, died from cardiac failure	*1/*3	AS: 1.0	(IM)	*1/*1	*1A/*1B	*1/*3	*1/*1	Metoprolol		
17	78	NSIP, died from cardiac failure	*1/*6	AS: 1.0	(IM)	*1/*1	*1A/*1B	*3/*3	*2/*2	Metoprolol		
18	90	NSIP, died from cardiac failure	*1/*6	AS: 1.0	(IM)	*1/*2	*1A/*1A	*3/*3	*1/*1			
19	77	NSIP, died from lung carcinoma	*1/*4	AS: 1.0	(IM)	*1/*1	*1A/*1A	*3/*3	*1/*1			
20	72	NSIP, no follow-up data yet	*1/*4	AS: 1.0	(IM)	*1/*3	*1A/*1A	*3/*3	*1/*1	Metoprolol		
21	79	IPF, no follow-up data yet	*4/*4	AS: 0.0	(PM)	*1/*1	*1A/*1A	*3/*3	*1/*1			
22	80	IPF, no follow-up data yet	*1/*5	AS: 1.0	(IM)	*1/*1	*1A/*1A	*3/*3	*1/*1			

# half of the dosage every other day; * part of the official notation of the allele combination that make-up the cytochrome P450 genotypes.

**Table 2:** CYP2D6 phenotype frequencies in the interstitial lung disease cases and healthy male volunteers [7].

CYP2D6 Phenotypes	Cases (n = 22)	Healthy Volunteers (n = 78)
Poor metabolizer	9 (41%)	8 (10.3%)
Intermediate metabolizer	13 (59%)	19 (24.4%)
Extensive metabolizer	0 (0%)	51 (65.3%)
Ultra-rapid metabolizer	0 (0%)	0 (0%)
